# COVID‐19 pandemic and impact on cancer clinical trials: An academic medical center perspective

**DOI:** 10.1002/cam4.3292

**Published:** 2020-07-10

**Authors:** Michelle Marcum, Nicky Kurtzweil, Christine Vollmer, Lisa Schmid, Ashley Vollmer, Alison Kastl, Kelly Acker, Shuchi Gulati, Punita Grover, Thomas J. Herzog, Syed A. Ahmad, Davendra Sohal, Trisha M. Wise‐Draper

**Affiliations:** ^1^ UC Cancer Center University of Cincinnati Medical Center Cincinnati OH USA; ^2^ Hematology and Oncology University of Cincinnati Cincinnati OH USA; ^3^ Department of Obstetrics and Gynecology University of Cincinnati Cancer Institute and College of Medicine Cincinnati OH USA; ^4^ Department of Surgery University of Cincinnati Cincinnati OH USA

**Keywords:** cancer, clinical trials, COVID‐19, oncology, pandemic

## Abstract

The COVID‐19 pandemic changed health‐care operations around the world and has interrupted standard clinical practices as well as created clinical research challenges for cancer patients. Cancer patients are uniquely susceptible to COVID‐19 infection and have some of the worst outcomes. Importantly, cancer therapeutics could potentially render cancer patients more susceptible to demise from COVID‐19 yet the poor survival outcome of many cancer diagnoses outweighs this risk. In addition, the pandemic has resulted in risks to health‐care workers and research staff driving important change in clinical research operations and procedures. Remote telephone and video visits, remote monitoring, electronic capture of signatures and data, and limiting sample collections have allowed the leadership in our institution to ensure the safety of our staff and patients while continuing critical clinical research operations. Here we discuss some of these unique challenges and our response to change that was necessary to continue cancer clinical research; and, the impacts the pandemic has caused including increases in efficiency for our cancer research office.

## Graphical abstract

The COVID‐19 pandemic has resulted in important changes in cancer clinical research operations and procedures. This commentary discusses some of these unique challenges and our response to change that was necessary to continue cancer clinical research; and, the impacts the pandemic has caused including increases in efficiency for our cancer research office.

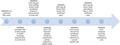

## 1 BACKGROUND

The novel severe acute respiratory syndrome coronavirus 2 (SARS‐CoV2) causes the respiratory disease, COVID‐19. It was first reported in December 2019 in China, spread rapidly throughout the world, and was designated as a pandemic in March 2020.^1^ COVID‐19 is associated with a range of presentations and can vary in severity from an asymptomatic infection to a lethal acute respiratory distress syndrome. Accumulating evidence suggests that patients with cancer are more susceptible to COVID‐19 than the general population. Among 1524 patients with cancer admitted to a single center in Wuhan, 12 (0.79%) patients were diagnosed with COVID‐19 compared to 0.37% in the community during the same time period.^2^ The WHO‐China joint commission reported a 7.6% case fatality rate in patients with cancer compared to 1.4% in those without any comorbid conditions suggesting worse outcomes in cancer patients.^3^ In a prospective cohort of 1590 patients, the 18 patients with a history of cancer were at increased risk of severe events compared with patients without cancer (39% vs 8%, *P* = .0003).^4^ These findings were further supported by Zhang et al who retrospectively identified 28 patients with cancer and COVID‐19 admitted to various hospitals in Wuhan, China and reported prevalence and mortality of COVID‐19 in cancer patients were 1.7 and 10 times higher, respectively, than that of the general population.^5^ Patients who received treatment for cancer within 14 days of COVID‐19 diagnosis were at a higher risk of severe events. Although these studies were limited by small sample sizes and heterogeneous populations with respect to histology, stage of cancer, and therapy,^6^ they provided early insight into the challenges of cancer care during this pandemic.

The oncology community face many difficult questions regarding the benefits of continuing cancer treatments against the risk of acquiring COVID‐19 from exposure to health‐care facilities and risks of complications from the disease. Health‐care delivery and oncology practice have had to adapt and transform in the span of a few weeks. Experience from other countries like Italy provided a broad framework for management of cancer patients and clinics to mitigate the risk of COVID‐19^7^ for which we adapted further.

In addition, the decision whether to continue cancer clinical trials became a safety concern regarding the treatment effect on cancer patients’ vulnerability for COVID‐19 contraction, potential viral exposure risk to clinical trial staff, and potential impact of an infected clinical trial cancer patient on study outcomes.^8^, ^9^

## 2 MANAGEMENT OF CANCER PATIENTS WITH COVID‐19 DIAGNOSIS

Guidelines on testing and management of patients with COVID‐19 evolved rapidly and continue to remain in flux. We attempted to stay at the leading edge as much as possible in guidance from the Governor of Ohio. Fortunately, the caseload in Ohio in general and the Greater Cincinnati area (our catchment area), in particular, remained lower than projected. Nonetheless, extensive monitoring, multidisciplinary input, logistical restructuring, and efficient communication of the decisions were required. Beginning March 15, COVID‐19 testing was offered. By April 15, ~1700 unique patients had been tested, and 124 (7.3%) had tested positive. Initially, an external laboratory was used, with a turnaround time of 6.8 days. Subsequently, we switched to an in‐house test, with a turnaround time of 1.6 days.

For our outpatient cancer clinic, we instituted strict guidelines on patient management. One day prior to appointment in clinic, patients were called and asked about COVID‐19 symptoms—fever, cough, shortness of breath, and flu‐like symptoms. If anyone answered yes, they were directed to an outpatient testing center. This was initially a drive‐through arrangement, and later transitioned to a dedicated outpatient setting. All patients coming into our outpatient cancer center building had temperature checks and COVID‐19 screening questionnaires administered. To date, during this screening and testing, 15 cancer patients, none of whom were on clinical trials, were found to be COVID‐19 positive.

As we learned more about COVID‐19 exposure and risks to our patients, we minimized exposure with televisits, mailing consent forms to patients, and shipping study drugs when possible. We also restricted family members and other non‐patient visitors. Additionally, we held treatments or spaced out infusion appointments. When patients presented to the cancer center for treatment, we requested them to wear masks based on randomized controlled trials confirming that mask use reduces transmission of COVID‐19.^10^

For those patients who continued to be seen for treatment, it was important to weigh the risk of observation or standard of care treatment vs clinical trial options. NCCN guidelines recommend a clinical trial when available. However, the risk of COVID‐19 infection created additional challenges. For example, patients with low‐risk disease on adjuvant therapy in the form of a clinical trial vs standard of care observation would be handled much differently than patients with high risk of disease progression. The latter cohort continued enrollment, while for the former, we recommended holding treatment. In metastatic patients, the same scenario exists. In patients with differentiated thyroid cancer, hormone‐sensitive prostate cancer, or similar in whom disease often progresses slowly, it was reasonable to forgo both standard of care treatment and clinical trials while patients with pancreatic cancer may have limited options and a clinical trial may potentially be life‐saving.

In addition, the type of treatment must also be considered. Cytotoxic chemotherapy often results in myelosuppression potentially putting cancer patients at increased risk of serious illness or even death if infected with COVID‐19.^2^ Cytotoxic chemotherapy has the potential to cause lymphopenia and Tan et al reported that lymphopenia was associated with poor prognosis (patients with lymphocyte percentage of < 5% in peripheral blood not only became critically ill but also sustained a high mortality rate).^11^ A report by Liang et al from China had previously reported that patients who received chemotherapy had a numerically higher risk of developing severe adverse events.^4^ These reports lead us to inquire as to whether certain measures, such as postponing adjuvant chemotherapy, would save some cancer patients from exposure to COVID‐19.

However, targeted agents without myelosuppression risk may provide better safety. Immunotherapy presented unique challenges as activating the immune system might prevent or mitigate infection,^12^ but subsequently could potentiate a cytokine storm in those with COVID‐19.

Therefore, a careful consideration of benefits and risks of standard of care therapy compared with clinical trials options must be performed for each individual patient. The COVID‐19 and Cancer Consortium (CCC19.org) representing almost 100 institutions worldwide is collecting data on these patients, which may provide insight into some of these considerations.^13^

## 3 COVID‐19 IMPACT ON CLINICAL AND RESEARCH OPERATIONS

**FIGURE 1:**
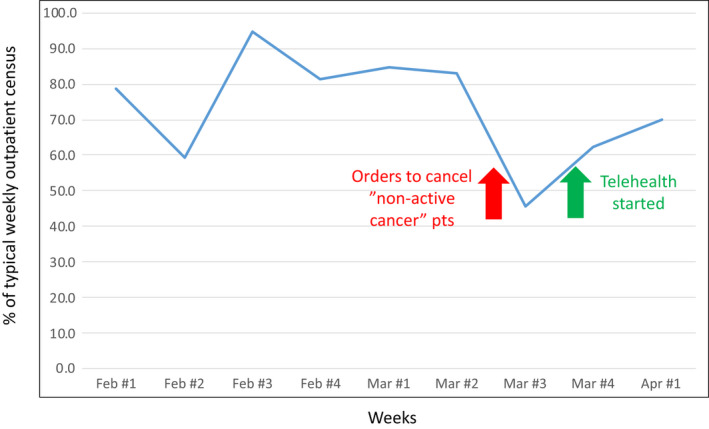
University of Cincinnati Center Patient Visit Volume During COVID‐19 Pandemic. Total ambulatory visits by week, from Feb 1 to Apr 4, 2020, for cancer diagnoses. Benchmark for 100% is the average during a typical week, first quarter of 2019

Universally, outpatient clinic volumes declined substantially during COVID‐19. Elective procedures were cancelled, non‐emergent evaluations were postponed, and many usual referrals were curtailed. Specific to cancer, second opinions were largely abandoned, in‐clinic personnel were limited, and usual multidisciplinary care was transitioned to virtual tumor board discussions. Since clinical volumes are the denominator for clinical trial enrollment, there is an expected direct impact of reductions in the former on the latter. At our institution, we sent out a directive on March 13 to decompress clinics to maximize physical distancing for patients as well as employees. Our policy was to switch to seeing only those patients who had active cancer management needs. “Active” cancer management was defined as: work‐up (patient evaluation, staging scans, and diagnostic/staging/therapeutic procedures) and treatment (chemotherapy, labs for treatment, and therapeutic trials) of cancer. Outpatient clinic volume during the third week of March was less than 50% of a typical week (Figure 1). Since patients continued to have clinical needs and questions, we rapidly instituted telehealth options—both video and phone visits—starting 23 March 2020. As can be seen in Figure 1, volumes (physical and telehealth combined) rapidly escalated back to around 70% of typical.

**FIGURE 2:**
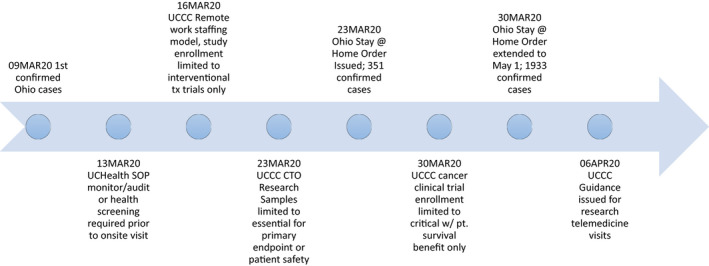
Clinical Research Operations Changes Timeline During COVID‐19. As state COVID‐19 cases increased, state orders were released and research operations changes were implemented either in advance or in real time

**FIGURE 3:**
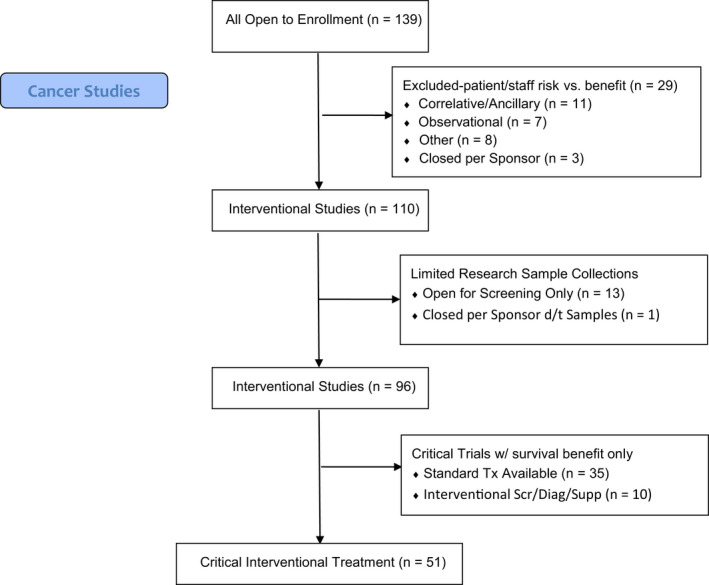
Cancer Clinical Trials Consort Flow Diagram. Flowchart of cancer studies open to patient enrollment. Continued reduction in available cancer trials as pandemic evolved and operations changed until only those most critical for cancer therapy remained

From a cancer clinical trials perspective, concerns for appropriate trial and patient management during the 2020 COVID‐19 pandemic revolved around limiting operations to critical functions only, while sustaining treatment administration capabilities using guidance from local and national IRBs, FDA, and cancer professionals’ organizations including American Society of Clinical Oncology (ASCO), American Association of Cancer Institutes (AACI), American College of Surgeons (ACS), and other associated subspecialty societies (See Timeline in Figure 2). Critical research was defined for our purposes as research that if discontinued, would endanger the lives of the human subjects participating in the research. As such, our response to emerging information was to temporarily halt enrollment to studies and clinical trials that were unlikely to affect participant survival or those that imposed unnecessary risk to staff. Specifically, noninterventional trials (observational and translational), interventional trials that did not involve treatment (supportive care, diagnostic, screening, and other), and interventional clinical trials involving treatment that did not meet our definition of critical research were closed to new patient enrollment (Figure 3). This required regular meetings of the management team and the Protocol Review and Monitoring Committee (PRMC) chair to review each open study as well as those in the pipeline. If an investigator disagreed with the decision, an appeal to the PRMC chair could be made. Interventional treatment clinical trials such as those in the setting of metastatic disease or early phase trials that offer cancer treatment options when no standard option exists remained open to new patients.

Several study sponsors elected independently to halt enrollment to their trial temporarily due to the pandemic. It is expected that trial enrollment numbers will decrease significantly during the COVID‐19 pandemic due to the closure of noninterventional and selected interventional trials. For the first 8 months of the fiscal year (July 2019‐Feb 2020), the noninterventional accruals averaged 31 per month. For the same time period, the interventional accruals averaged 40 per month. For the month of March 2020, when decisions to halt trials were rolled out, the noninterventional accrual was 15 and interventional accrual was 29. The enrollment numbers are expected to decline further while restrictions are in place.

For our externally sponsored trials, study sponsor visits, such as Site Initiation Visits and Interim Monitoring Visits, were conducted entirely remotely. The change required swift steps in infrastructure support on behalf of the institution to allow access to electronic medical records (EMRs) for study monitoring purposes. With the advent of COVID‐19 travel restrictions, the University of Cincinnati Cancer Center (UCCC) Clinical Trials Office (CTO) was well positioned to continue with existing remote monitoring of sub‐site EMRs, regulatory and pharmacy source documents, and we have utilized the ability of the REDCap database for electronic data capture to identify and resolve data quality items remotely. For sub‐sites unable to conduct remote monitoring activities, monitoring schedules have been re‐adjusted to allow for flexibility in the timing of on‐site monitoring pending the lifting of travel restrictions.

With respect to research regulatory documentation, the UCCC CTO Regulatory department was well positioned to adapt to many of the operational changes during the COVID‐19 pandemic. Due to the nonclinical nature of the work, moving to a remote model still allowed for consistency in most workflows and processes. The UCCC CTO had adopted an electronic regulatory system in 2019 that was 21 CFR Part 11 compliant, allowing regulatory staff and monitors to maintain access to the majority of regulatory binders remotely, via a web‐based platform. Because IRB submissions were done electronically, there was no disruption in IRB, PRMC, or data safety monitoring board (DSMB) activities. Similarly, all FDA submissions were transitioned to electronic submissions to allow for continuity of work. The shift to an at‐home work model required the use of electronic signatures for many items that were previously provided as wet‐ink. Changes in clinical care due to COVID‐19 have also affected regulatory work, as many of these changes implemented for patient safety required deviating from study protocols. The IRBs utilized by UCCC CTO released guidance regarding the reporting of these COVID‐19 related deviations allowing for these deviations to be considered not promptly reportable and for immediate changes in study procedures to promote patient safety to not require preemptive IRB approval.

Physicians and staff continued to conduct study patient visits per protocol as long as the visits required safety labs and study medications. However, some changes such as allowing evaluation of treatment tolerability and functional status by phone, and routing adverse events, safety labs, and procedures in the EMR to the investigators for electronic signatures allowed clinical coordinators to work remotely. Study patient visits that did not involve a medically necessary procedure (ie, follow‐up visit, questionnaire, and protocol visit where treatment is not involved) were rescheduled, conducted remotely, or not completed. Similar to clinical monitoring of patients, both clinical trial patients and employees were screened for COVID‐19 symptoms. In addition, employees were instructed to self‐monitor for fever prior to leaving home for work and provided masks to wear for the duration of their workday.^14^

In addition to telemedicine visits, remote informed consent practices were developed. With IRB approval, physicians and staff could conduct the consent process with potential participants via phone. A blank copy of the consent form was mailed or emailed to the patient with an accompanying letter and staff contact information. Potential participants called and discussed the form with the study team, and if the individual elected to participate, they signed their copy, scanned, and emailed to the study team followed by mailing the wet‐ink signature version. The study team signs upon receipt and follows usual practices. The entire process is thoroughly documented in the participant record.

Collection and processing of correlative research samples that did not affect patient safety or the primary endpoint of the clinical trial were also temporarily halted. For this purpose, correspondence regarding the halt and an accompanying inquiry were sent to the study sponsor regarding the feasibility of continuation of study participation or new enrollment. Sponsor response was documented and tracked for each clinical trial and this deviation from normal study procedures was reported to the IRB.

## 4 OUTCOMES

To date, no study participants have discontinued active protocol treatment during the pandemic for reasons outside of standard such as disease progression. More importantly, there have not been any clinical trial participants diagnosed with COVID‐19 at our institution as of 23 April 2020.

We have submitted several COVID‐19 related reportable events for the IRB’s review and approval, but their guidance has allowed for efficiency in allowing a single reportable event to cover multiple potential events for a study when the event was the same for multiple participants. The FDA, our IRBs of record, and our UCCC CTO all provided early guidance for continuity of clinical trials, and have provided ongoing guidance, which has allowed for organized and consistent regulatory operations. It is likely that the impact of the COVID‐19 pandemic will continue to be seen in regulatory work for the near future. We may also see a lasting shift in how much of our regulatory work is completed, as a more remote model may provide solutions to physical office space issues, and efficiencies not previously realized.

The overall impact of operational changes on quality can be assessed more fully after the COVID‐19 restrictions have been lifted and metrics from the UCCC clinical trials management system and internal auditing activities from this time period can be reviewed. It is anticipated that there will be an increase in protocol deviations due to changes made to protocol activities to mitigate immediate apparent risks to subjects. The UCCC CTO has maintained standardized practices to ensure study documentation is in compliance with FDA and GCP standards and to allow for adequate PI oversight remotely. The CTO rapidly created and released new workflows to allow for the remote review and sign‐off on study activities such as eligibility, clinical significance, and remote consent procedures. Internal auditing and monitoring practices have continued during this time through review of the part 11 compliant e‐regulatory system and EMR review of source documentation with an extended timeframe for corrections by the study team. Additional resources have been devoted to developing continuing education initiatives implemented remotely such as video recordings of training and onboarding activities (eg, mock consent and AE review). We anticipate these remote education resources will be a valuable contribution to the CTO and will continue to be created/utilized after the COVID‐19 restrictions are lifted. Many of the changes that the UCCC CTO made in order to adjust to the challenging time of the COVID‐19 pandemic resulted in greater efficiency of our staff and physicians, evidence that even in a time of despair and much personal difficulty, innovation can emerge.

## AUTHOR CONTRIBUTIONS

Michelle Marcum, Nicky Kurtzweil, Christine Vollmer, Lisa Schmid, Ashley Vollmer, Alison Kastl, Kelly Acker, Shuchi Gulati, Punita Grover, Thomas J. Herzog, Syed A. Ahmad, Davendra Sohal, Trisha M. Wise‐Draper: the above authors contributed via conceptualization, data curation, writing ‐ original draft, and writing ‐ review and editing.

## Data Availability

The data that support the findings of this study are available from the corresponding author upon reasonable request.
